# The germline-enriched Ppp1r36 promotes autophagy

**DOI:** 10.1038/srep24609

**Published:** 2016-04-21

**Authors:** Qinghua Zhang, Maomao Gao, Ying Zhang, Ying Song, Hanhua Cheng, Rongjia Zhou

**Affiliations:** 1 Department of Cell Biology, Wuhan 430072, P. R. China; 2 Department of Genetics, College of Life Sciences, Wuhan University, Wuhan 430072, P. R. China

## Abstract

Spermatogenesis is a highly regulated process during which haploid sperm cells are generated. Although autophagy is involved in the spermatogenesis process, the molecular pathways and regulations of autophagy in germ cell development remain elusive. Here, we showed that Ppp1r36, a regulatory subunit of protein phosphatase 1, is expressed during gonadal development, mainly in testes during spermatogenesis. Autophagy protein LC3 (microtubule associated protein 1 light chain 3), especially its active form LC3-II, had a similar expression pattern to Ppp1r36. Moreover, LC3-II level and puncta analysis showed that autophagy is up-regulated around 21 dpp (day postpartum) in postnatal testis, indicating a potential role of autophagy during the first wave of spermatogenesis. We demonstrated that Ppp1r36 promotes autophagosome formation upon starvation induction. Further autophagy flux analysis using a tandem fluorescent indicator, mCherry-GFP-LC3, confirmed that Ppp1r36 participated in autophagy. We further determined that Ppp1r36 is associated with Atg16L1 (autophagy related 16-like 1) in autophagy of starvation induction. Thus, our results uncover a potential role of the regulatory subunit Ppp1r36 of protein phosphatase 1 in enhancing autophagy during spermatogenesis.

In mammals, the primordial germ cells migrate into the genital ridge, which then differentiate into gonads during embryogenesis. Spermatogenesis is a complexly regulated process during which haploid sperm cells are generated from spermatocytes through meiosis in the seminiferous tubules in male testis^1^. The haploid round spermatids first appear around postnatal day 18 and further undergo a series of dramatic morphological transformations and structural changes, such as the nuclei condensed and cytoplasm discharged, as well as the formations of flagella and acrosome. Finally, elongated sperm cells are generated in the seminiferous tubules in approximately 35 days and a new round of spermatogenesis is initiated about every 12 days^2^,^3^,^4^,^5^.

Spermatogenesis is a developmental process, which is involved in both mitosis and meiosis. Nearly half of total coding genes are expressed in mouse testis^6^, which are localized on both sex-chromosomes and autosomes. The Y-linked genes are particularly involved in spermatogenesis. For example, *Azf* (Azoospermia factor) mutations can cause sertoli cell-only syndrome and spermatogenic arrest^7^, *Daz* (Deleted in azoospermia) mutations lead to oligozoospermia and azoospermia^8^,^9^, and *Zfy2* is responsible for sperm formation^10^. Some X-linked genes are also required for spermatogenesis in mammals. For example, the *Dax1*-deficiency in mice results in spermatogenic failure^11^. Many autosomal genes are essential to spermatogenesis, of which *Dmrt1* is a key transcriptional gatekeeper that controls the mitosis versus meiosis decision in male germ cells^12^,^13^. Endocrine hormones regulate coordinately spermatogenesis, such as follicle stimulating hormone and luteinizing erythropoietin^5^. It is suggested that the GnRH (gonadotropin-releasing hormone) pulse generator is a master ON/OFF switch of the hypothalamus-pituitary-gonad reproductive axis^5^. In addition, small non-coding RNA pathways are involved in regulations of spermatogenesis in mammals. Ablation of *Dicer*, which is required for microRNAs biogenesis in Sertoli cells, leads to a complete absence of spermatozoa and progressive testicular degeneration^14^. In contrast to miRNAs, piRNAs are enriched in germ line, which play their roles through their interactive proteins including Mili and Miwi. Their interactions are important in inhibition of transposable elements, translation regulation and RNA degradation during spermatogenesis^15^,^16^,^17^.

Accumulating evidence shows the importance of interplay between subcellular activities and germ cell development. Dynamic mitochondrial fusion is essential for spermatogenesis. Germ cell-specific gene *Gasz* contains a mitochondrial localization signal, which interacts with *Mfn1* to promote mitofusion^18^, indicating a role of mitofusion during germ cell development. Autophagy, as an important degradation and recycling pathway, can transport the intracellular components such as the excess or dysfunctional proteins to the lysosomes for degradation to maintain metabolism homeostasis^19^. Autophagy-related proteins (Atg) are highly conserved from yeast to mammals. Knockout of *Atg7* in germ cells resulted in irregular or nearly round-headed spermatozoa in mice, which is similar to human globozoospermia^20^, highlighting an importance of autophagy in germ cell development. However, roles and underlying molecular mechanisms of autophagy during spermatogenesis remain elusive.

Protein phosphatase 1 (PP1) is one of the main members of the serine/threonine protein phosphatase family which are ubiquitously expressed in eukaryotic cells^21^. It regulates a variety of cellular functions through interaction of its catalytic subunit with different established or putative regulatory subunits^21^,^22^,^23^. PP1 has multifaceted cellular functions in many physiological and pathological processes such as metabolism, immune response, apoptosis, mitosis, meiosis, protein synthesis and cytoskeletal reorganization^23^,^24^,^25^,^26^. A recent study shows that PPP1 inhibited autophagy in cardiomyocytes through Atg16L1 dephosphorylation, indicating a role of Atg16L1 phosphorylation in autophagy in cardiomyocytes^27^. Particularly, deletion of the *Ppp1cc* in germ cells resulted in oligo-terato-asthenozoospermia and male infertility in mice^28^. *Ppp1cc* mutant males had predominantly round spermatids and only occasional mature sperms visible in the epididymis, eventually led to male sterility, which is similar to a human condition known as nonobstructive azoospermia^29^. Recently, other PP1 isoforms including PPP1CB, PPP4C and PPP6C have also identified in human sperm^30^. In addition, sperm motility is initiated by inhibiting PP1 by Wnt signaling^31^. These studies indicate that protein phosphorylation is relevant to sperm physiology. However, regulations of protein phosphatase 1, especially with regard to the importance and underlying molecular mechanisms in germ cell development, remain largely unknown.

In the present study, we identify a regulatory subunit of protein phosphatase 1, Ppp1r36, in mouse gonads and show that Ppp1r36 and LC3 are associated with spermatogenesis in mice. Furthermore, we demonstrate that Ppp1r36 promotes autophagy upon starvation induction, probably through its interaction with Atg16L1. Thus, these results uncover a potential role for the regulatory subunit Ppp1r36 of protein phosphatase 1 in enhancing autophagy during spermatogenesis.

## Results

### Ppp1r36 is expressed in testes during spermatogenesis

**Figure 1:**
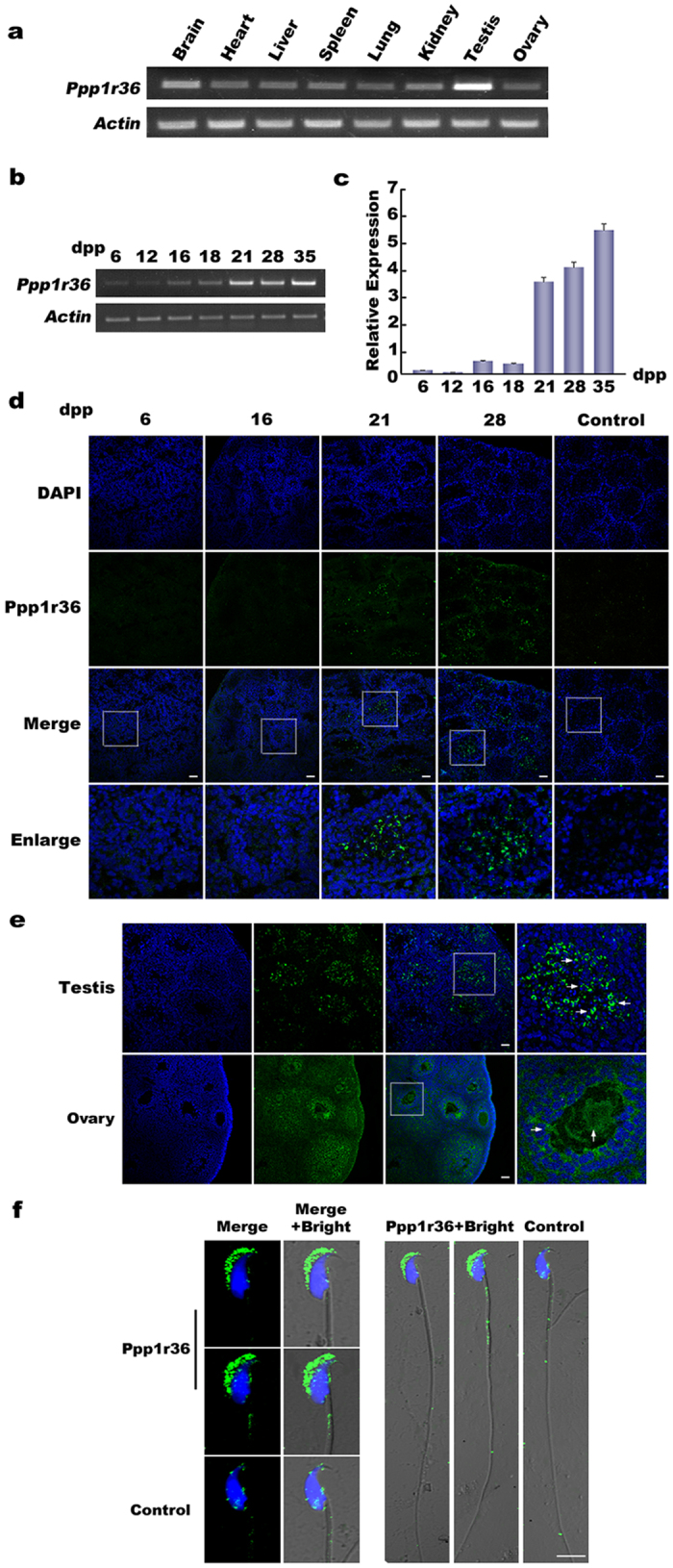
Ppp1r36 expression pattern in mice. (**a**) *Ppp1r36* mRNA expression in adult tissues in mice. RT-PCR was used to analyze the expression. *Actin* was used as an internal control. (**b,c**) *Ppp1r36* expression analysis in postnatal testes in mice by semi-quantitative RT-PCR (**b**) and real-time RT-PCR (**c**). (**d**) Immunofluorescence analysis of Ppp1r36 protein in postnatal testes in mice. Anti-Ppp1r36 and FITC-conjugated goat anti-rabbit IgG (H + L) antibodies were used to detect Ppp1r36 (green). The nuclei were detected by DAPI (blue). Preimmune serum was used as a negative control. Square areas in the inset were enlarged and showed in the bottom panel. Scale bar, 50 μm. Ppp1r36 positive signals were observed in spermatids after 21 dpp. (**e**) Immunofluorescence analysis of Ppp1r36 protein in adult testis and ovary. Ppp1r36 was detected by anti-Ppp1r36 and FITC-conjugated goat anti-rabbit IgG (H + L) (green). The nuclei were detected by DAPI (blue). Square areas in the inset were enlarged and showed on the right. Ppp1r36 positive signals were observed in spermatids and spermatozoa in testis, and oocytes and granulosa cells in ovary (arrows). Scale bar, 50 μm. (**f**) Immunofluorescence detection of Ppp1r36 protein in spermatozoa from epididymis of adult male mice. Ppp1r36 protein is expressed in the acrosome (green). The nuclei were detected by DAPI (blue). Preimmune serum was used as a negative control. Scale bar, 5 μm.

*Ppp1r36* is an evolutionarily conserved gene in vertebrates (Suppl. Fig. 1). To explore a potential role of *Ppp1r36* gene in spermatogenesis, we first investigated its expression patterns in mice. In adult tissues, *Ppp1r36* was highly expressed in testis compared with other tissues (Fig. 1a). During testis development in postnatal mice, *Ppp1r36* expression increased to a high level at 21 dpp and then had a stable level until adulthood (Fig. 1b,c).

Further immunofluorescence analysis showed that Ppp1r36 protein can be detected in testis at 21 dpp. With the progress of development, Ppp1r36 presented higher expression level until adulthood (Fig. 1d). Ppp1r36 positive signals were observed in spermatids and spermatozoa in testis, as well as in oocytes and granulosa cells in ovary (Fig.1e). In spermatozoa from epididymis of adult male mice, Ppp1r36 protein was mainly distributed in the acrosome (Fig. 1f). These data suggested that Ppp1r36 is associated with spermatogenesis in mice.

### LC3 is expressed in testes during spermatogenesis

**Figure 2:**
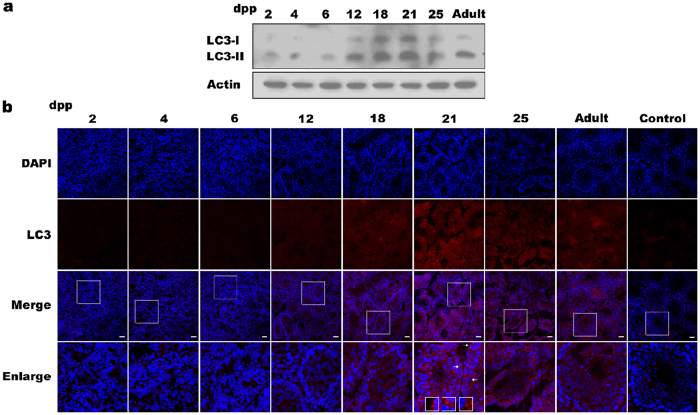
LC3 protein expression in postnatal and adult testes in mice. (**a**) Western blot analysis of LC3-I and LC3-II (active LC3 lipidation form) at different stages of postnatal testes. Actin was used as an internal control. (**b**) Immunofluorescence analysis of LC3 protein at different stages of postnatal testes using anti-LC3 and Cy3-conjugated goat anti-rabbit IgG (H + L) antibodies (red). The nuclei were detected by DAPI (blue). Square areas in the inset were enlarged and showed in the bottom panel. Some LC3 positive puncta were observed at 21 dpp (arrows). Scale bar, 50 μm.

A previous study showed a role of autophagy protein Atg7 in acrosome biogenesis in mice^20^, indicating that autophagy is important for spermatogenesis. We thus detected autophagy marker LC3 in mouse testes at different developmental stages. Western blot analysis showed that LC3 reached a high level of expression at 18–21 dpp. Notably, LC3-II (active LC3 lipidation form) had a highest abundance at the 18–21 dpp compared to other stages (Fig. 2a), indicating occurrence of autophagy during the first wave of spermatogenesis.

Immunofluenrence analysis further confirmed the observation (Fig. 2b). LC3 can be detected in testes from 18 dpp to adult, mainly in the cytoplasm of spermatocytes, spermatids and spermatozoa. Some LC3 positive puncta were observed at 21 dpp (Fig. 2b). These results suggested a potential role of autophagy during spermatogenesis.

### Ppp1r36 over-expression promotes autophagy upon starvation induction

**Figure 3:**
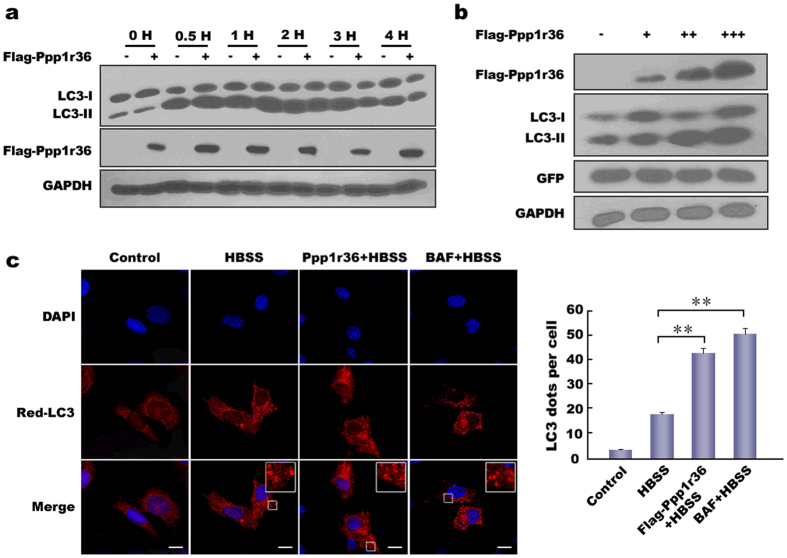
Ppp1r36 over-expression promotes autophagy upon starvation induction. (**a**) *Ppp1r36* over-expression up-regulates LC3-II level at the early stage of starvation induction. HEK293T cells were transfected with equal amounts of Flag-Ppp1r36 (+) or vector pRK-Flag (−) and cultured in the HBSS medium for 0, 1, 2, 3 and 4 hrs respectively. Cell lysates were analysed by immunoblotting with the indicated antibodies. GAPDH was used as an endogenous control. (**b**) LC3-II is up-regulated by *Ppp1r36* over-expression in a dose-dependent manner. HEK293T cells were transfected with gradient amounts of Flag-Ppp1r36 (0, 0.3, 0.6 and 0.9 μg) and equal amount of pEGFP-N1 (control). pRK-Flag was added for an equal amount DNA in each well. Western blot was used to detect the levels of LC3 after HBSS starvation for 1 h. GFP and GAPDH were used as endogenous controls. (**c**) *Ppp1r36* over-expression promotes autophagosome formation. GC-1 cells were co-transfected with Red-LC3 and Ppp1r36 or control vector pRK-Flag and cultured in the normal or HBSS medium for 1 h. In the BAF + HBSS group, the cells were treated with BAF for 3 hrs to inhibit autophagosome fusion with lysosome. The nuclei were counterstained with DAPI (blue). The images were taken by confocal microscopy. Square areas in the inset were enlarged to show LC3 puncta (red). Scale bar, 10 μm. LC3 puncta per cell were quantified and analyzed by *t*-test. Data are presented as means ± S.D. **p < 0.01.

To explore whether Ppp1r36 participates in autophagy, *Ppp1r36* was over-expressed in HEK293T cells and then the cells were treated in starvation medium to induce autophagy. Western blot analysis showed that LC3-II level was higher when *Ppp1r36* transfected upon starvation induction for 0.5–1 h compared to controls (Fig. 3a). We then confirmed the results by gradient transfection of *Ppp1r36* (Fig. 3b).

Fluorescence analysis in mouse germ cell GC-1 showed that the number of Red-LC3 puncta was significantly increased in *Ppp1r36* over-expression upon starvation induction compared to the cells without *Ppp1r36* transfection (Fig. 3c). In addition, BAF (Bafilomycin A1, a blocker of the formation of autolysosome) treatment also markedly increased the puncta numbers in comparison with controls. These results indicated that Ppp1r36 promotes autophagosome formation upon starvation induction.

### Autophagy flux associated with Ppp1r36

**Figure 4:**
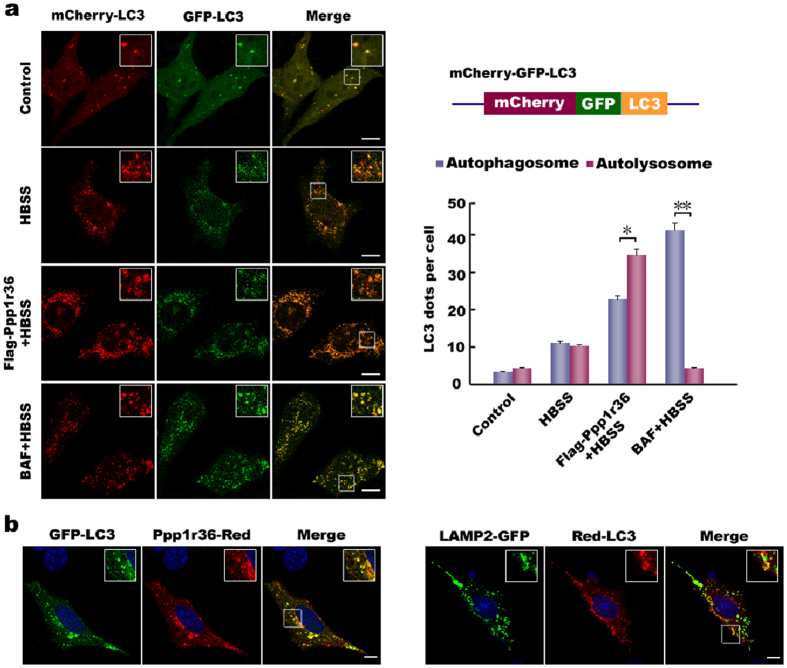
Autophagy flux associated with Ppp1r36. (**a**) Detection of autophagy flux. GC-1 cells were co-transfected with a tandem expression vector mCherry-GFP-LC3 and Flag-Ppp1r36 or control vector pRK-Flag, and cultured in normal or HBSS medium for 2 hrs. In the BAF + HBSS group, the cells were treated with BAF for 4 hrs to inhibit the fusion between autophagosome and lysosome. Images were analyzed by confocal microscopy. Square areas in the inset were enlarged and showed on the upper right. Yellow or green puncta indicate autophagosomes, while red puncta include autophagosomes and autolysosomes, because GFP protein is sensitive and attenuated in an acidic environment of autolysosome. Scale bar, 10 μm. The right panels show the tandem structure of mCherry-GFP-LC3 and the statistic analysis of LC3 puncta per cell by *t*-test. Data are presented as means ± S.D. *p < 0.05; **p < 0.01. (**b**) Ppp1r36 co-aggregates with LC3 puncta. GC-1 cells were co-transfected with Ppp1r36-Red and GFP-LC3, and LAMP2-GFP and Red-LC3 respectively. The cells were starved in the HBSS medium for 2 hrs. The nuclei were counterstained with DAPI (blue). Images were captured by confocal microscopy. Square areas in the inset were enlarged and showed at the upper corner. Marked co-aggregation dots were observed between Ppp1r36-Red and GFP-LC3, and LAMP2-GFP and Red-LC3. Scale bar, 10 μm.

To investigate autophagy process involved in Ppp1r36, we tested autophagy flux by analysis of a tandem fluorescent indicator, mCherry-GFP-LC3. Since green fluorescence of the fusion protein is very sensitive to the acidic environment of lysosomes and quickly quenched in autolysosomes, just red fluorescence could be detected in the autolysosomes^32^,^33^. Fluorescence analysis using the indicator system in GC-1 cells showed that *Ppp1r36* over-expression promoted autophagy flux upon starvation induction, while BAF treatment inhibited the process (Fig. 4a). In addition, Ppp1r36-Red could co-aggregate with GFP-LC3 puncta in GC-1 cells upon starvation induction (Fig. 4b). These results further confirmed that Ppp1r36 promotes autophagy.

### Ppp1r36 interacts with Atg16L1

**Figure 5:**
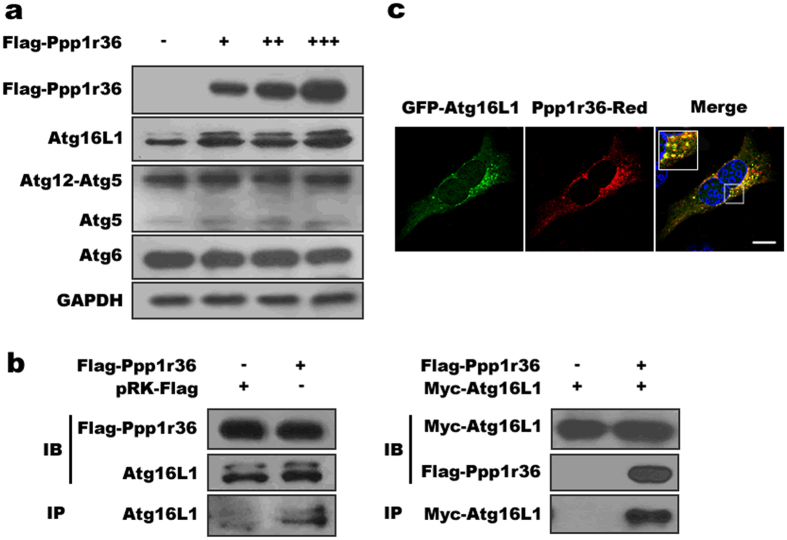
Ppp1r36 interacts with and up-regulates Atg16L1. (**a**) *Ppp1r36* over-expression up-regulates Atg16L1 protein level. HEK293T cells were transfected with gradient amounts of Flag-Ppp1r36 (0, 0.3, 0.6 and 0.9 μg) or pRK-Flag (control). The cells were cultured in the HBSS medium for 1 h. Western blot was used to detect the levels of Atg6, Atg12-Atg5 and Atg16L1. GAPDH was used as an endogenous control. (**b**) Co-immunoprecipitation analysis shows Ppp1r36 interaction with both endogenous and transfected Atg16L1. HEK293T cells were transfected with Flag-Ppp1r36 or empty vector (left) and Flag-Ppp1r36 and Myc-Atg16L1 (right) respectively, and cultured in HBSS medium for 2 hrs before harvest. For co-immunoprecipitation assays, the lysates were immunoprecipitated with an anti-Flag antibody followed by immunoblotting with an anti-Atg16L1 (left) or anti-Myc (right) antibody. (**c**) Co-localization between Atg16L1 and Ppp1r36 puncta. GC-1 cells were co-transfected with Ppp1r36-Red and GFP-Atg16L1 and cultured in HBSS medium for 1 h. The nuclei were counterstained with DAPI (blue). The images were taken by confocal microscopy. Square areas in the inset were enlarged and showed on the upper left. Scale bar, 10 μm.

To further illustrate the molecular mechanism of Ppp1r36-associated autophagy, we tested levels of autophagy related proteins in autophagosome formation. Western blot analysis showed that endogenous Atg16L1 protein level increased with an increasing amount of Ppp1r36, while protein levels of Atg6 and Atg12-Atg5 did not change (Fig. 5a). Co-immunoprecipitation analysis indicated an interaction of Ppp1r36 with Atg16L1 (Fig. 5b). Further fluorescence analysis showed that Ppp1r36 and Atg16L1 puncta were co-aggregated in GC-1 cells (Fig. 5c). These results indicated that Ppp1r36 was associated with Atg16L1.

## Discussion

Both kinases and phosphatases cooperatively control protein activities through phosphorylation/dephosphorylation. Ppp1c is ubiquitously expressed in nearly all tissues. Cellular and tissue specificity of dephosphorylation by Ppp1c are dependent on its regulatory subunits. Hence, search for the interactive proteins that regulate Ppp1c activity is essential to understand signaling pathways of autophagy regulation, especially in germ cell development. In the present study, we identify a regulatory subunit of protein phosphatase 1, Ppp1r36, in mouse gonads.

The study presents several major findings: (i) Ppp1r36 is expressed during gonadal development, especially in testes during spermatogenesis. (ii) Interestingly its expression pattern is consistent with autophagy key protein LC3. (iii) Moreover, autophagy is up-regulated around 21 dpp in postnatal testis, revealed by LC3-II level and puncta, an active form of LC3, indicating a potential role of autophagy during the first wave of spermatogenesis. (iv) To explore a possible association of Ppp1r36 with autophagy, we show that Ppp1r36 promotes autophagosome formation upon starvation induction. (v) Further autophagy flux analysis using a tandem fluorescent indicator, mCherry-GFP-LC3, confirms that Ppp1r36 promotes autophagy. (vi) Lastly, we show that Ppp1r36 can interact with Atg16L1. It has been shown that Atg16L1 interacted with Atg12-Atg5 to promote LC3 lipidation by acting as an E3-like ubiquitin ligase^34^,^35^. Thus, our results uncover a potential role for the regulatory subunit Ppp1r36 of protein phosphatase 1 in enhancing autophagy during spermatogenesis.

To date, nearly 40 genes for serine⁄threonine phosphatases have been identified^36^. Many interaction proteins of PP1 are detected, for example, 77 proteins in human testis and 7 proteins in human sperm that bind PP1 have been identified^36^. This is consistent with the fact that cellular and tissue specificity of dephosphorylation by PP1 are dependent on its regulatory subunits in various tissues. Ppp1r36 has been identified as an interactive protein of PP1 by a global proteome analysis^37^. We show that as a regulatory subunit, Ppp1r36 probably exerts its role in spermatogenesis through autophagy. The process is probably dependent on its interaction with Atg16L1. A recent study indicated that PPP1 inhibited autophagy in cardiomyocytes through Atg16L1 dephosphorylation, suggesting a role of Atg16L1 phosphorylation for autophagy induction in cardiomyocytes^27^.

Ppp1r36 is not only expressed in the developing testis during the first wave of spermatogenesis, but also presents in the acrosome of mature spermatozoa, indicating a role of Ppp1r36 in sperm activity, probably through autophagy. Previous study using gene chip (Affymetrix) exhibited a differential expression pattern of Ppp1r36 in XX and XY SSCs (spermatogonial stem cells) with a higher level in XY than XX gonad of E12.5^38^. Accumulating evidences show that spermatogenesis process is involved in autophagy. Specific knockout of *Atg7* in mouse germ cells resulted in irregular or nearly round-headed spermatozoa, which is similar to human globozoospermia, thus the knockout mice were infertile because of the defection of acrosome biogenesis^20^. Heat stress can induce autophagy in addition to apoptosis in mouse germ cells^39^. In deed, autophagy plays a role in the survival of spermatozoa during conservation in refrigeration^40^. Autophagy marker LC3-II and autophagosomes were also detected in primary cultures of spermatocytes isolated from male rats^41^. Recent study showed that *Ol-epg5* (ectopic P-granules autophagy protein 5 homolog, a new critical component of the autophagy pathway) knockout in medaka fish resulted in an impaired autophagic flux^42^. Taken together, these data suggest that autophagy is probably involved in several processes of spermatogenesis from the first wave of spermatogenesis to mature of spermatozoa. Nevertheless, the molecular mechanisms of autophagy in spermatogenesis still need further investigation.

## Methods

### Ethics statement

All animal experiments and methods were performed in accordance with the relevant approved guidelines and regulations, as well as under the approval of the Ethics Committee of Wuhan University.

### Antibodies and reagents

Primary antibodies: Anti-Atg5 (Cat# AP1812b) was purchased from Abgent, San Diego, USA. Anti-Atg6 (Cat# PD017) was from MBL, Nagoya, Japan. Anti-Atg16L1 (Cat# 8089) was purchased from Cell Signaling Technology, Pickering, Canada. Anti-GAPDH (glyceraldehyde-3-phosphate dehydrogenase, Cat# CW0100) was from CWBIO, Beijing, China. Anti-Actin (Cat# 14395-1-AP) was from Proteintech Group, Chicago, USA. Anti-Flag antibody (Cat# F3165) and monoclonal anti-LC3 (Cat# SAB4200361) were from Sigma-Aldrich, St Louis, USA. Anti-Myc (Cat# 11667149001) and anti-GFP (Cat# 11814460001) were from Roche Applied Science, Indianapolis, USA. Anti-Ppp1r36 was prepared by Beijing Huada Protein Innovation, Beijing, China.

Secondary antibodies: Goat anti-mouse IgG (H + L), horseradish peroxidase conjugated antibody (Cat# 31430) and goat anti-rabbit IgG (H + L), horseradish peroxidase conjugated antibody (Cat# 31460) were from Pierce Company, Rockford, USA. FITC-conjugated immunopure goat anti-rabbit IgG (H + L) (Cat# ZF-0311) was purchased from Feiyi Technology, Wuhan, China. Cy3-conjugated affinipure goat anti-rabbit IgG (H + L) (Cat# SA00009-2) was from Proteintech Group, Chicago, USA.

### Cloning of *Ppp1r36* and RT-PCR

Total RNAs from mouse tissues were isolated using the Trizol Reagent (Cat# 15596-026, Invitrogen, Carlsbad, USA). We synthesized cDNA by reverse transcription from the total RNAs with MMLV reverse transcriptase (Cat# M1701, Promega, Madison, USA). Full-length *Ppp1r36* (NM_001163103.1) was cloned into pRK-Flag using *Sal*1 and *Not*1 (Fermentas, Lithuania) to generate Flag-Ppp1r36 and into pDsRed-N1 using *Xho*I and *Bam*HI (Fermentas) to Ppp1r36-Red. Primers were as follows:

Ppp1r36-*Sal*1: 5′-acgcgtcgaccatggtcaagagtgaggccatgttcacc-3′

Ppp1r36-*Not*1: 5′-aaggaaaaaagcggccgctttagggcaggtgcttggcgaag-3′

Ppp1r36-*Xho*1: 5′-ccgctcgagatggtcaagagtgaggccatgttc-3′

Ppp1r36-*Bam*H1: 5′-cgcggatccgctttagggcaggtgcttggcg-3′

The underlined letters indicated restriction endonuclease sites.

For RT-PCR analysis, cDNA synthesis was as described above. *Ppp1r36* and *Actin* were amplified with an annealing temperature of 60 °C for 36 cycles (*Ppp1r36*) and 25 cycles (*Actin*) respectively. Primers for the amplification were:

*Ppp1r36*-1: 5′-agaaaggcaagaaagggaa-3′

*Ppp1r36*-2: 5′-cgtggtgaaggagcagat-3′

*Actin*-1: 5′-actgtgcccatctacgaggg-3′

*Actin*-2: 5′-gtggtggtgaagctgtagcc-3′.

### Immunofluorescence analysis

Cells and tissue sections were fixed in pre-cooled 100% methanol at −20 °C or 4% paraformaldehyde at room temperature for 30 min respectively, 0.1% Triton X-100/PBS permeabilized for 10 min and then blocked in 5% bovine serum albumin/PBS for 30 min at 37 °C. The samples were incubated with anti-Ppp1r36 polyclonal antibodies in 5% bovine serum albumin/PBS overnight at 4 °C. Then the samples were incubated with FITC-conjugated affinipure goat anti-rabbit IgG for 1 h at room temperature. Nuclear staining was carried out with DAPI Staining Solution (Cat# C1002, Beyotime Institute of Biotechnology, Jiangsu Province, China) for 10 min in the dark. Images were captured with a confocal fluorescence microscope (Olympus, FV1000, Tokyo, Japan).

### Cell culture, treatment and transfection

HEK293T and GC-1 cells were cultured in DMEM (Cat# SH30022.01B, HyClone, Beijing, China) with 10% FBS (fetal bovine serum, Cat# SV30087.02, HyClone). The Cells were transfected in 12/24-well plates using Lipofectamine 2000 (Cat# 11668027, Invitrogen) according to the routine protocol. For starvation treatment, the cells were cultured in the medium HBSS (Cat# SH30030.02B, HyClone). For BAF treatment, Bafilomycin A1 (Cat# B1793, Sigma-Aldrich) was added in the medium for 3 hrs before harvest.

### Western blot analysis

HEK293T cells transfected with expression vectors were lysed in RIPA lysates (50 mM Tris pH7.4, 150 mM NaCl, 0.5% NP40, 1 mM EDTA, 1% Protease inhibitors). Cocktail (Roche) was used as protease inhibitor. After centrifuged for 10 min at 4 °C, the supernatant was used for Western blot analyses. Western blots were performed according to routine protocols. The protein extracts from HEK293T cells or mouse tissues were separated using 15% SDS-PAGE and transferred onto a 0.45 μm PVDF membrane (Cat# NK0414, Roche Diagnostics, Indianapolis, USA). The membranes were blocked with 5% non-fat dried milk in TBST (20 mM Tris-HCl, pH7.5, 150 mM NaCl, 0.1% Tween-20) and incubated with the antibodies overnight at 4 °C, followed by the horseradish peroxidase-conjugated secondary antibody. The protein bands were visualized by incubating membranes with the ECL Plus detecting reagents (Cat# WBKLS0500, Millipore, Billerica, USA).

### Co-immunoprecipitation assays

To analyze protein interactions, co-immunoprecipitation assays were performed in HEK293T cells. The cells were lysed in RIPA lysates as above. The cell lysates were incubated with the anti-Flag antibody and Protein G Agarose (Roche) overnight at 4 °C. The resins were collected by centrifugation at 4 °C and then washed four times with RIPA lysates. Bound proteins were eluted using loading buffer (3% SDS, 1.5% mercaptoethanol, 8% glycerol, 0.01% Coomassie blue G-250, 150 mM Tris-HCl pH7.0) and separated using 15% SDS-PAGE, followed by immunoblotting with anti-Atg16L1 or anti-Myc antibody.

## Additional Information

**How to cite this article**: Zhang, Q. *et al.* The germline-enriched Ppp1r36 promotes autophagy. *Sci. Rep.* **6**, 24609; doi: 10.1038/srep24609 (2016).

## Supplementary Material

Supplementary Information
