# Supplementary material for: The germline-enriched Ppp1r36 promotes autophagy
Source: Sci Rep. 2016 Apr 21;6:24609. doi: 10.1038/srep24609 (PMC4838909; doi:10.1038/srep24609)
Supplement: Supplementary Information [file srep24609-s1.pdf]

Supplementary Information for

The germline-enriched Ppp1r36 promotes autophagy

Qinghua Zhang[1], Maomao Gao[2], Ying Zhang[2], Ying Song[1], Hanhua Cheng[1*], Rongjia Zhou[2*]

[1]Department of Cell Biology & [2]Department of Genetics, College of Life Sciences, Wuhan University, Wuhan 430072, P. R. China

*Corresponding authors: Professors Rongjia Zhou and Hanhua Cheng, College of Life Sciences, Wuhan University, Wuhan 430072, P. R. China, Fax: 0086-27-68756253, E-mail: rjzhou@whu.edu.cn, hhcheng@whu.edu.cn

This file includes:
Figure S1

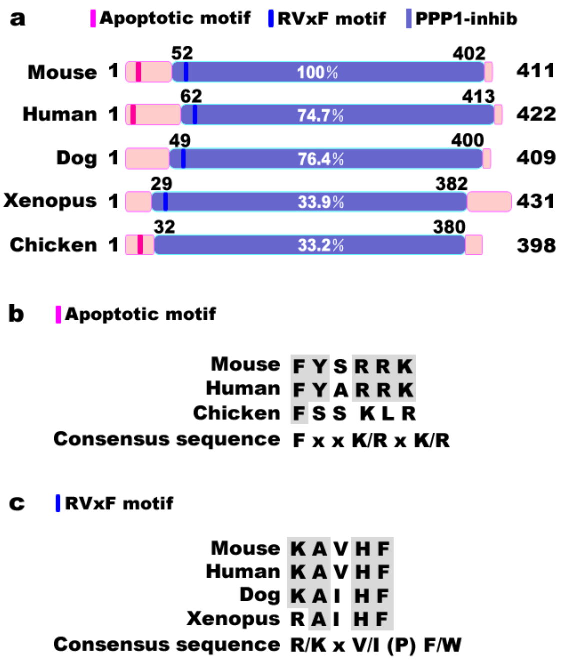

Figure S1 | Evolutionary conservation of Ppp1r36 protein in vertebrates. (a) Ppp1r36 protein has an RVxF and/or an apoptotic motif and a PPP1-inhib domain. GenBank access numbers are, NP_001156575.1 (mouse), NP_758953.1 (human), XP_004936481.1 (chicken), XP_013971444.1 (dog) and NP_001088688.1 (*Xenopus*). (b) Alignment of the apoptotic motif in Ppp1r36 of mouse, human and chicken. The consensus sequence, F-x-x-K/R-x-K/R. (c) Alignment of the RVxF motif in Ppp1r36 of mouse, human, dog and frog. The consensus sequence, R/K-x(0,1)-V/I-(P)-F/W, where x can be any residue, present or absent and (P) refers to any residue but proline.
